# Microbial community in resistant and susceptible Churra sheep infected by *Teladorsagia circumcincta*

**DOI:** 10.1038/s41598-022-21058-x

**Published:** 2022-10-21

**Authors:** Verónica Castilla Gómez de Agüero, Cristina Esteban-Blanco, Héctor Argüello, Elora Valderas-García, Sonia Andrés, Rafael Balaña-Fouce, Juan José Arranz, Beatriz Gutiérrez-Gil, María Martínez-Valladares

**Affiliations:** 1 Instituto de Ganadería de Montaña, CSIC-Universidad de León, 24346 Grulleros, León, Spain; 2 Departamento de Sanidad Animal, Facultad de Veterinaria, Universidad de León, 24071 León, Spain; 3 Departamento de Ciencias Biomédicas, Facultad de Veterinaria, Universidad de León, 24071 León, Spain; 4 Departamento de Producción Animal, Facultad de Veterinaria, Universidad de León, 24071 León, Spain

**Keywords:** Microbial communities, Parasitology

## Abstract

Gastrointestinal nematodes (GIN) are a major threat to health and welfare in small ruminants worldwide. *Teladorsagia circumcincta* is a nematode that inhabits the abomasum of sheep, especially in temperate regions, causing important economic losses. Given that *T. circumcincta* and microbiome share the same niche, interactions between them and the host are expected. Although it is known that within a sheep breed there are animals that are more resistant than others to infection by GIN, it is not known if the microbiome influences the phenotype of these animals. Under this condition, 12 sheep were classified according to their cumulative faecal egg count (cFEC) at the end of a first experimental infection, 6 as resistant group (RG) and 6 as susceptible group (SG) to *T. circumcincta* infection. Then, all sheep were experimentally infected with 70,000 L3 of *T. circumcincta* and at day 7 days post-infection were euthanized. At necropsy, gastric mucosa and gastric content from abomasum were collected to extract bacterial DNA and sequence V3-V4 region from 16S rRNA gene using Ilumina technology. After bioanalysis performed, results showed that α-diversity and β-diversity remained similar in both groups. However, resistant phenotype sheep showed a higher number of bacteria butyrate-fermenting species as *Clostridium sensu stricto 1* (abundance in RG: 1.29% and in SG: 0.069%; *p* = 0.05), and *Turicibacter* (abundance in RG: 0.31% and in SG: 0.027%; *p* = 0.07) in gastric content but also *Serratia* spp in gastric mucosa (abundance in RG: 0.12% and in SG: 0.041%; *p* = 0.07). A trend towards a significant negative correlation between cFEC and *Clostridium sensu stricto 1* abundance in gastric content was detected (r = − 0.537; *p* = 0.08). These data suggest that microbiome composition could be another factor associated with the development of the resistant phenotype modifying the interaction with the host and the in last instance affecting the individual risk of infection.

## Introduction

Infections caused by gastrointestinal nematodes (GIN) are one of the most important diseases in grazing ruminants in temperate regions of the world^1–3^. The economic importance of these infections is related to reduced weight gain, milk and meat production and reproduction; it was estimated that GIN infection could have cost to European Union-28 €2.1 bn in 2018^4,5^. However, in order to determine if microbiota influences GIN infection control, in this study we have described the composition and diversity of the microbiome in Churra breed sheep classified as resistant or susceptible to the infection by the GIN *Teladorsagia circumcincta*. *T. circumcincta* is the most prevalent nematode species present not only in the area where ewes were selected for the present study but also in many other temperate areas of the world^6–8^. For that, the microbiome presents in gastric mucosa and gastric content from 12 ewes experimentally infected with *T. circumcincta* have been sequenced and characterized. Besides, prevalence have suffer un increase since 1990 in the region this study was carried on^9^. GIN infections have been regularly controlled with anthelmintic drugs, mainly with bezimidazoles due to their good quality-cost relationship, combined with pasture management^8,10^. But, these drugs were incorrectly applied along the years -overused, misused, or applied incorrectly- causing the appearance of anthelmintic resistance (AR) and favoring the survival of parasites with resistant genotype^11^. Since in 1960s the first report about benzimidazole resistance was published, many farms has been informed as resistant; besides, last year farms with multi-resistant parasites are becoming increasing common^12–15^. This situation has generated the need to change the approach for GIN control with new strategies such as the development of vaccines against helminths, biological control with anthelmintic active compounds from plants and fungus, pasture management or the development of breeding programs for worm resistance^11,16–18^.

Under this circumstance, the interest in selecting animals with resistant phenotype to GIN infection, especially sheep, has increased within last years^19^ defined resistance as the ability of a host to suppress the establishment and/or subsequent development of a roundworm infection. Therefore, according to this concept, authors described resistant phenotype as those host that present low worm burden despite grazing pastures contaminated with infective larva. The most frequent method to measure the parasite burden is counting the number of worm eggs in faeces, being expressed as the faecal egg counts (FEC)^20^.

Besides, it has been shown that there are sheep breeds that are more resistant to GINs infection than other breeds, such as Canaria Hair, Barbados Blackbelly and Red Maasai, but at the same time within a particular breed there are individuals that are more resistant than others, such as within the Churra breed^19,21–23^. Genetic resistance to GIN have been associated with a protective immune response that is mediated by humoral (titre of antibodies, mainly IgA and IgE) and cellular response (eosinophils, T cells, globule leukocytes or mast cells)^23–26^. However, these studies have not considered an important component of the host that is in direct contact with gastrointestinal parasites and therefore could influence the worm burden, the gastrointestinal microbiota.

All mammals are colonized by wide diversity of microorganisms that live in symbiosis in the mucosal surfaces of the host ^27,28^. Microbiota produces a beneficial relationship host-bacteria, providing nutrients, metabolizing them and defending against colonization by pathogens^29,30^. However, occasionally harmful organisms, such as GIN, colonize the gastrointestinal tract provoking damages whose severity depends to the burden of the infection, the localization or the parasite specie, among others^31^. As GIN and bacteria share the habitat, it is expected that interact among them and with the host. Although it is known that parasitic infections are associated with profound changes in the structure and function of the host gut microbiome, in veterinary medicine this knowledge is limited and most of the studies have been conducted in human and rodent models^27,31–33^.

Understanding the composition of microbiome in presence of GIN is fundamental to develop a better knowledge about the pathogenesis of the infection. However, in order to determine if microbiota influences GIN infection control, in this study we have described the composition and diversity of the microbiome in Churra breed sheep classified as resistant or susceptible to the infection by the GIN *T. circumcincta*. For that, the microbiome present in gastric mucosa and gastric content from 12 ewes experimentally infected *with T. circumcincta* have been sequenced and characterized.

## Results

### Total bacteria DNA

The number of copies in gastric mucosa was 100-1300 and between 840,000–9,000,000 for gastric content without significant differences between groups.

### Taxonomic profile analysis and microbiota

The sequencing of the V3-V4 region of the 16S rRNA gene performed for the 12 sheep gastric mucosa samples generated an average of 1,169,865,8 raw reads, while the gastric content generated a total of 231,472.6 raw. After removing host genome contamination, we retained an average of 413,853.9 sequences for abomasal mucosa and 174,316.8 sequences for gastric content. Retained reads were used for the subsequent analyses. Then the DADA2 analysis performed for the 12 ewes analyzed in this work identified 11,217 ASVs for abomasal mucosa and 8,514 ASVs for gastric content. The sampling depth was set to 1500.

In total, 23 phyla were identified in both types of samples, 1 from Archaea domain and 22 from Bacteria domain. The most abundant phyla in gastric mucosa, representing approximately 96% of the total, were Bacteroidetes (48.23%), Firmicutes (29.60%), Actinobacteria (9.27%), Verrucomicrobia (2.72%), Proteobacteria (2.50%), Fibrobacteres (2.26%), and Spirochaetes (2.20%). No significant differences were found in phyla abundances between resistant and susceptible groups in these gastric mucosa samples. While, in gastric content the most abundant phyla, which accounted approximately 94%,were Bacteroidetes (43.15%), Firmicutes (28.60%), Actinobacteria (14.13%), Fibrobacteres (2.89%), Spirochaetes (2.35%), Kiritimatiellaeota (2.06%) and Proteobacteria (1.38%). Differences approaching significance (*p* = 0.09) were found in the gastric content for Actinobacteria phylum between resistant and susceptible animals, showing an abundance of 10.2% in RG and 18.2% in SG.

**Figure 1:**
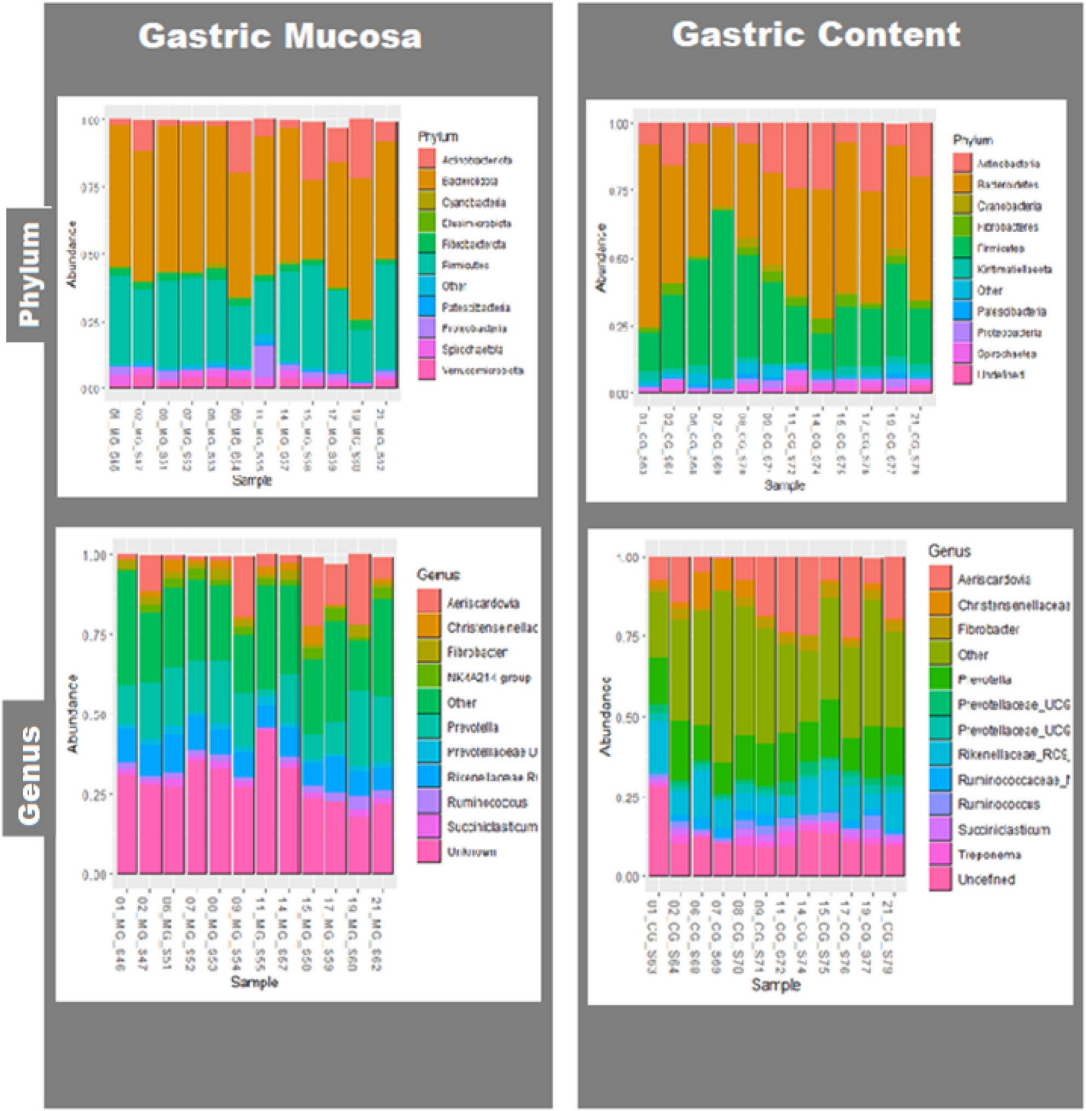
Relative percentage of abundance for the main taxa for phylum and genus level in resistant and susceptible groups.

**Figure 2:**
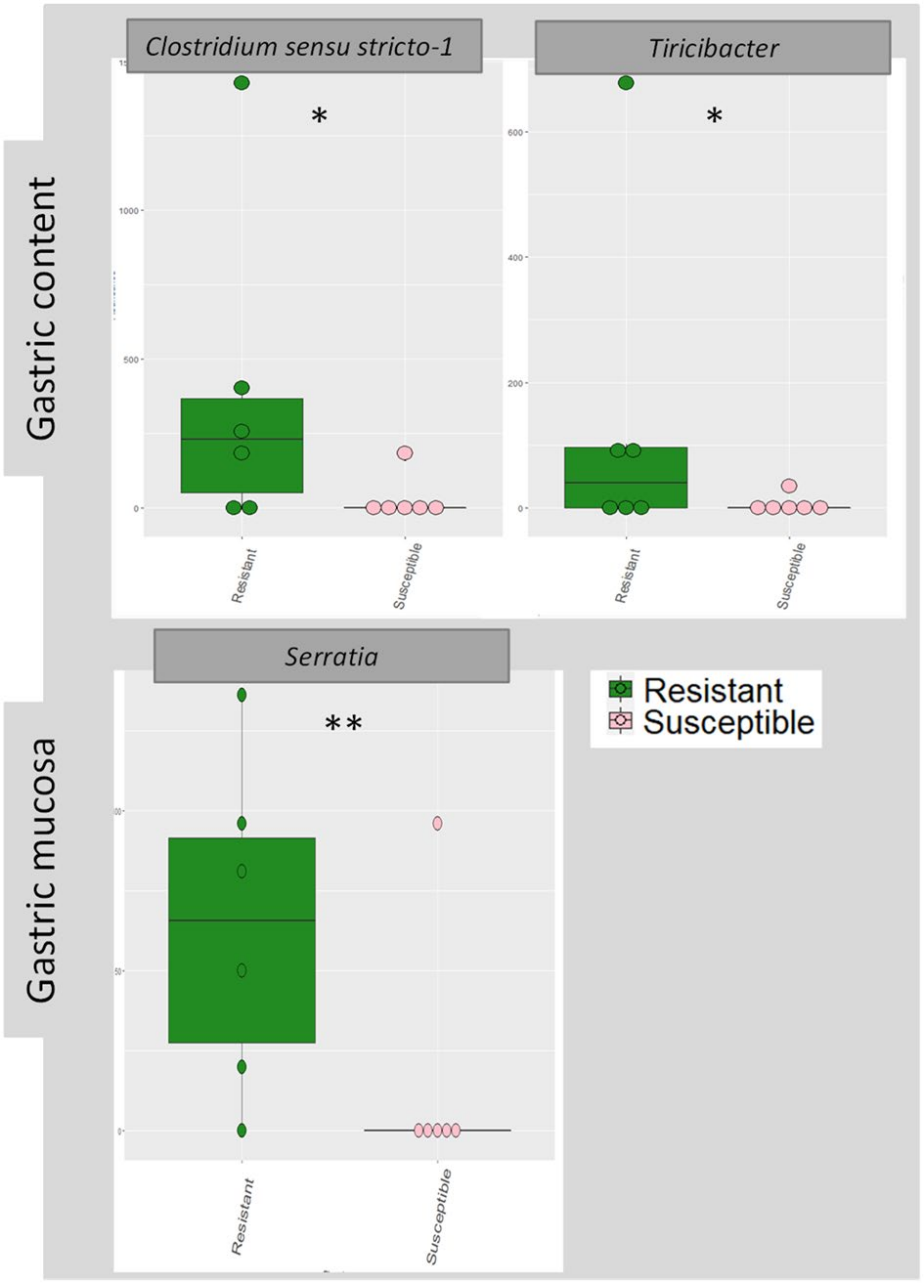
Boxplot showing significant or slight significant differences for RG and SG in gastric content (*Clostridium sensu stricto 1;* *p* = 0.08 and *Turicibacter*; *p* = 0.07), and gastric mucosa (*Serrata* spp ; *p* = 0.05). Significant differences are indicated by ** *(p* < 0.05) and slight significant differences by * (*p* between 0.05–0.01).

**Figure 3:**
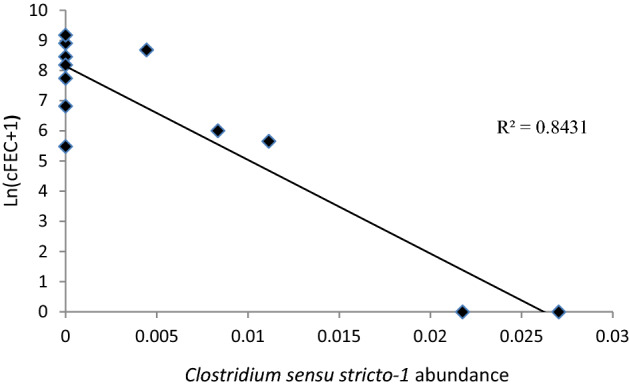
Slight significant negative correlation between cFEC at the end of the first infection and *Clostridium sensu stricto 1* abundance. Rho =− 0.537 and *p* = 0.08.

The most abundant genus in gastric mucosa was *Prevotella* with accounted 15%, followed by *Rikenellaceae* RC9 (8.96%) and *Aeriscardovia* (8.70%) among other. In gastric content, *Prevotella,* with 14% of abundance, was followed by *Aeriscardovia* (13.42%) and *Rikenellaceae* RC9 (8.17%). Regarding the differences in genus abundance between groups, *Serratia* spp genus showed differences approaching significance (*p* = 0.07; RG 0.12% and SG 0.041%) in gastric mucosa. In gastric content, significant differences were found for *Clostridum sensu stricto-1* (*p* = 0.05; RG 1.29% and SG 0.069%) and close to significance for *Turicibacter* (*p* = 0.07; RG 0.31% and SG 0.027%). The Spearman correlation coefficient between cFEC measured at the end of the first infection and the abundance of *C. sensu stricto-1* (*r*= − 0.537; *p*= 0.08) in gastric content showed an approaching significance negative correlation; no correlations whereas found for other species between *Serratia* spp and cFEC (Table 1, Figs. 1, 2, 3).

**Table 1:** Principal taxa in gastric mucosa and gastric content samples between RG and SG in *T. circumcincta* sheep.infected.

	Phylo	Clase	Order	Family	Gender	Without groups (%)	RG (%)	SG (%)
Gastric Mucosa					*Prevotella*	15.01	15.70	14.30
					*Prevotella *UCG. 001	1.76	1.75	1.87
					*Prevotella *NK3B31 Group	0.59	0.49	0.70
				Prevotellaceae	*Prevotella* UCG.003	1.66	1.65	9.11
					*Rikenellaceae RC9* gut groups	8.96	8.80	9.11
	Bacteroidota	Barterodia	Bacteroidales	Rikenellaceae	SP3-e08	1.40	0.71	2.19
	Actinobacteria	Actinobacteria	Bifidobacteriales	Bifidobacteriaceae	*Aeriscardovia*	8.71	7.19	10.22
					*Butyrivibrio*	0.73	0.81	0.65
				Lachnospiraceae	*Pseudobutyrivibrio*	0.50	0.52	0.48
				Ruminococcaceae	*Ruminococcus*	1.94	2.00	1.88
				Christensenellaceae	*Christensenella R7*	1.93	2.16	1.70
				Acidaminococcaceae	*Succiniclastrum*	1.8	1.94	1.74
				Clostridiaceae	*Clostridicum sensu stricto-1*	0.87	1.26	0.48
			Clostridiales	Veilonellaceae	*Quinella*	0.59	0.59	0.59
					NK4A214 group	2.49	2.62	2.32
					UCG-005	1.29	1.49	1.08
	Firmicutes	Clostridia	Oscillospirales	Oscllospiraceae	*Papillibacter*	0.95	0.82	1.09
	Fibrobacteres	Fibrobacteria	Fibrobacterales	Fibrobacteraceae	*Fibrobacter*	2.22	2.16	2.27
	Spirochaetes	Spirochaetes	Spirochaetales	Spirochaetaceae	*Treponema*	1.59	1.85	1.32
					*Prevotella*	14.00	15.00	13.00
					*Prevotella* UCG. 001	2.20	2.17	2.47
				Prevotellaceae	*Prevotella* UCG.003	2.08	2.17	1.99
	Bacteroidota	Barterodia	Bacteroidales	Rikenellaceae	*Rikenellaceae RC9* gut groups	9.67	10.40	8.95
				Christensenellaceae	*Christensenella R7*	3.10	3.48	2.71
					*Ruminococcus*	1.81	1.68	2.02
Gastric Content					NK4A214 group	1.70	1.58	2.05
				Ruminococcaceae	*Ruminococcus UCG-014*	1.39	1.63	1.13
		Clostridia	Clostridiales	Clostridiaceae	*Clostridicum sensu stricto-1*	0.61	1.14	0.70
	Firmicutes	Negativicutes	Acidaminococcales	Acidaminococcaceae	*Succiniclastium*	1.65	1.46	1.90
	Actinobacteria	Actinobacteria	Bifidobacteriales	Bifidobacteriaceae	*Aeriscardovia*	13.55	9.55	17.55
	Fibrobacteres	Fibrobacteria	Fibrobacterales	Fibrobacteraceae	*Fibrobacter*	2.85	2.78	2.93
	Spirochaetes	Spirochaetes	Spirochaetales	Spirochaetaceae	*Treponema*	1.87	1.98	1.92

### Alpha and Beta diversity

**Figure 4:**
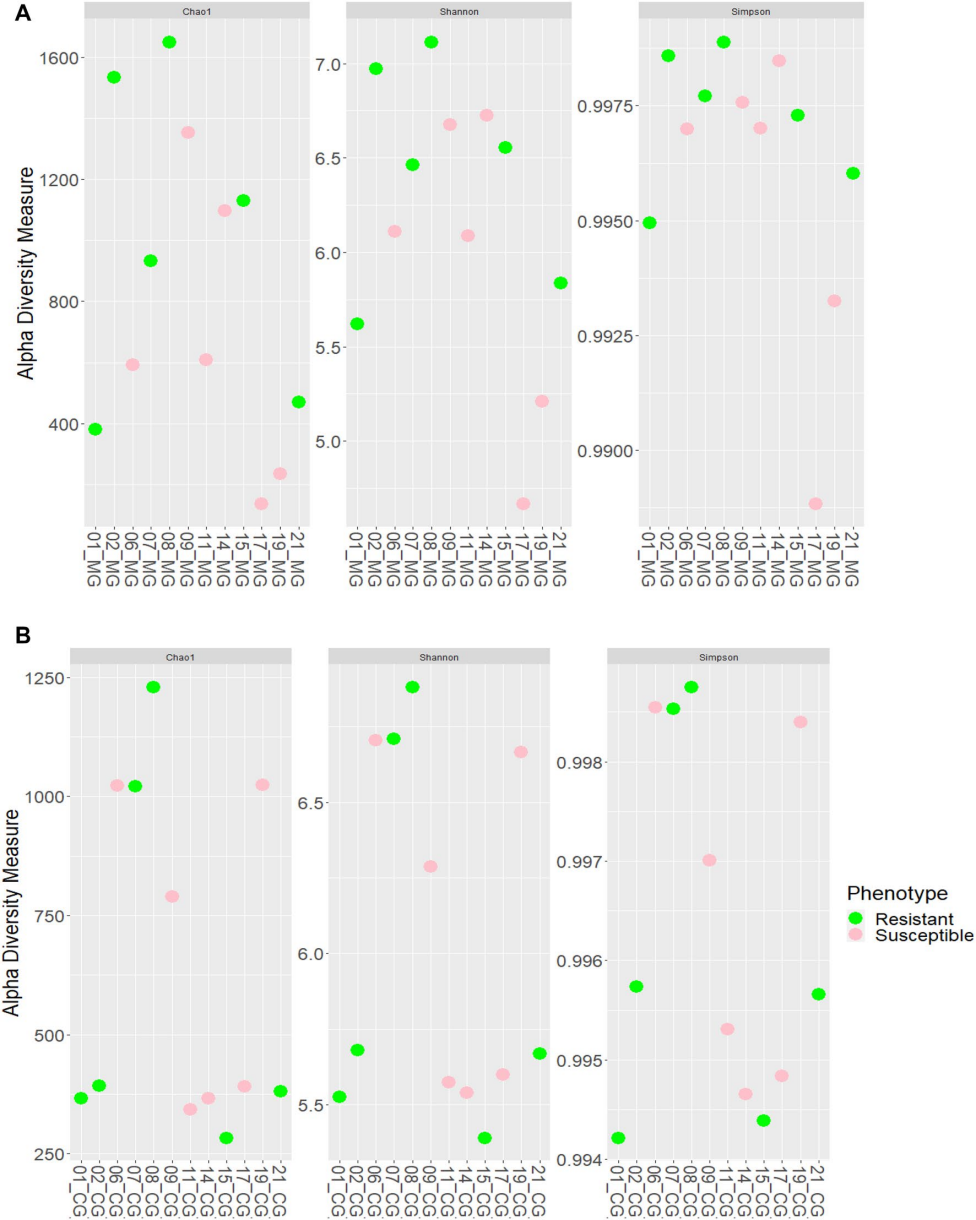
Estimation of richness (Shannon and Chao indexes) and evenness (Simpson index) in gastric mucosa (**A**) and gastric content (**B**) in RG (green) and SG (pink) experimentally infected with *T. circumcincta.* Not significance differences detected between groups.

No significant differences between resistant and susceptible animals were observed in alpha diversity by any of the estimators used in the study. However, the gastric mucosa presented *p* values lower than gastric content (Tables 2, 3 and Fig. 4) (Chao index: *p* = 0.260; Shannon index: *p* = 0.243; and Simpson index: *p* = 0.271). Beta-analysis showed a trend to clustering for resistant animal samples from gastric mucosa; however no trend was detected for gastric content.

**Table 2:** Estimation of evenness and richness (Chao, Shannon and Simpson indexes) in gastric mucosa and gastric content samples during *T. circumcincta* infection*.* No significant differences between resistant and susceptible animals were observed.

Sample_ID	Phenotype	Type of Sample	Chao 1	Shannon	Simpson
1	Resistant	Gastric Mucosa	380.000	5.619	0.995
2	Resistant	Gastric Mucosa	1534.286	6.971	0.999
6	Susceptible	Gastric Mucosa	592.167	6.109	0.997
7	Resistant	Gastric Mucosa	933.625	6.462	0.998
8	Resistant	Gastric Mucosa	1648.625	7.114	0.999
9	Susceptible	Gastric Mucosa	1351.964	6.677	0.998
11	Susceptible	Gastric Mucosa	609.000	6.088	0.997
14	Susceptible	Gastric Mucosa	1097.000	6.723	0.998
15	Resistant	Gastric Mucosa	1131.000	6.556	0.997
17	Susceptible	Gastric Mucosa	137.000	4.668	0.989
19	Susceptible	Gastric Mucosa	236.000	5.208	0.993
21	Resistant	Gastric Mucosa	470.167	5.836	0.996
1	Resistant	Gastric Content	365.429	5.525	0.994
2	Resistant	Gastric Content	392.000	5.679	0.996
6	Susceptible	Gastric Content	1023.000	6.705	0.999
7	Resistant	Gastric Content	1020.333	6.708	0.999
8	Resistant	Gastric Content	1229.038	6.880	0.999
9	Susceptible	Gastric Content	790.000	6.286	0.997
11	Susceptible	Gastric Content	342.000	5.575	0.995
14	Susceptible	Gastric Content	366.000	5.541	0.995
15	Resistant	Gastric Content	283.000	5.390	0.994
17	Susceptible	Gastric Content	390.231	5.599	0.995
19	Susceptible	Gastric Content	1024.176	6.664	0.998
21	Resistant	Gastric Content	380.000	5.668	0.996

**Table 3:** *p* values between RG and SG for each estimator and each sample.

Variables	Estimator		
	Chao1	Shannon	Simpson
Differences RG versus SG (gastric mucosa)	0.260	0.243	0.271
Differences RG versus SG (gastric content)	0.872	0.870	1.000

The datasets used and/or analyzed during the current study available from the corresponding author on reasonable request.

## Discussion

This study characterizes for first time the microbiome composition in gastric mucosa and content of Churra breed ewes with resistant and susceptible phenotype to the infection by *T. circumcincta*. The aim was to determinate if the microbiota could influence the resistant phenotype to the infection by *T. circumcincta* in Churra sheep. Animals were classified as resistant or susceptible according to their cFEC during a first experimental infection but also confirmed by IgA levels against L4 *T. circumcincta* by^23^. In that study, these resistant ewes showed higher IgA levels in serum at day 3 post-infection (*p* < 0.05) and close to significance at day 21 pi (*p* = 0.06); moreover, a strong negative correlation between cFEC and specific IgA was only significant in resistant ewes at day 3 pi (r =− 0.870; *p* < 0.05), but absent in susceptible ones. Several studies in different breed sheep have reported the association between high levels of specific IgA against the GIN and lower worm burden and FEC^21,34^.

Microbial diversity is evaluated using α-diversity indices such a richness or abundance of species from each community. Dissimilarities between communities are studied using β-diversities based on their ordination. In our study, neither gastric content microbiome nor gastric mucosa microbiome showed significant differences between groups in any α-diversity indices studied. On the contrary of our data^35^, reported significant differences in various microbial alpha-diversity indices between infected and non-infected group at 7 days post infection (dpi) by *Haemonchus contortus*. These differences could be due to all the animals of our study were infected^36^ studied abomasal microbiota composition in naive and immune calves infected with *Ostertagia ostertagi* to described and understand mechanism related with protective immunity. The authors suggested that unlike naive animals, the *O. Ostertagi* infection in immune cattle induced a minimal disruption in the abomasal microbiota and this may contribute to the development of long-term protective immunity.

In recent years, several studies have focused on the impact that GINs produced on host’s microbiome composition and how affects the parasite establishment and control of the infection comparing non-infected and infected animals^37–39^. It have been reported that GINs infections involve a change in the structure in the host digestive microbiome, inducing different physiological changes depending on the parasite specie or host^29,37,38,40–42^. The microbiome composition of abomasum from non-infected sheep is mainly represented by Bacteroidetes (≈65%) and Firmicutes (≈25%) phyla, but 7 days post-infection with *H. contortus*, Bacteroidetes increased (≈71%) and Firmicutes decreased (≈18%)^35^. In our study, Bacteroidetes (RG 46%; SG 49%) abundance in gastric mucosa was higher than Firmicutes (RG 33%; SG 25%) for both groups. Nevertheles, SG showed higher Bacteroidetes: Firmicutes ratio although differences were not statistically significant, probably because all animals were infected, as stated above^43^ sequenced gastric content from Merino sheep naturally infected with H. contortus field strains reporting higher relative abundance in Firmicutes (45%) than Bacteroidetes (26%). These results differ with most of studies and our own results, where Bacteroidetes (RG 46%; SG 40%) was more abundant than Firmicutes (RG 30%; SG 26%). Authors explained these variations in microbiome composition by factors that affect microbiome, as parasite burden, breed type, diets and different environmental condition. Besides, the relative abundance of Actinobacteria phylum in gastric content, which represented the 14.13% of the total bacteria, showed slight differences between resistant (10.2%) and susceptible (18.2%) sheep.

At genus level, *Prevotella* has been described as the most affected by GINs infections caused by *H. contortus*, *Trichostrongylus colubriformis* and *T. circumcincta* in sheep and goats. An increase in relative abundance in obligate anaerobes taxa, as Prevotellaceae family in lambs, sheep and goats infected with *H. contortus, T. colubriformis* and *T. circumcincta* was detected in infected animals compared with non-infected animals^44–47^. It could be explain by the *Prevotella*role in protein degradation and energy host metabolism compensation^44,48^. Although in this study *Prevotella* was the most abundant genus, 15% in gastric mucosa and 14% in gastric content, no differences were shown between resistant and susceptible sheep in none sample presumably because all animals were infected.

Butyrate is a short-chain fatty acid (SCFA) that is formed during the microbial fermentation of dietary fiber of ruminants. This metabolite is present in low concentrations, and it seems to be involved not only in nutrition, but also as a potent inhibitor of intestinal inflammation^47^detected a decrease in metabolic pathway genes related to butyrate after an infection with H. contortus and *T. circumcincta* in lambs. Besides, this decrease in butyrate metabolism was following by the abundance reduction in some butyrate-producers bacteria species. On the other hand^44^, supposed that nematode infection modulates the gut butyrate biosynthesis by altering the abundance of butyrate-producing bacteria and they detected significant differences between non-infected and *H. contortus* infected goats in the relative abundance of the genus *Butyrivibrio* in rumen, which is a bacteria butyrate producer. In our study, differences were not found in *Butyrivibrio* genus in gastric content, but we found significant differences others butyrate-producers bacteria as *C.sensu stricto-1* (RG 1.29% and SG 0.069%) and close to the significance limit with *Turicibacter* (RG 0.31% and SG 0.027%) between RG and SG in gastric content, been RG which account higher percentage^49^. Besides, this microorganism showed an approaching significant negative correlation with cFEC (*r*= − 0.537; *p*= 0.08). This data support the hypothesis that ewes with lower FEC have higher abundance of C. *sensu strict-1*.

The production of natural compounds with nematicidal activity synthesized by microorganisms is being a new focus in GINs infections chemical control investigation^50^ demonstrated that *Serratia* spp produces volatile compounds with 100% in vitro nematicidal activity against plant nematodes^51^ tasted in vitro isolated chitinases produced by Serratia sp. against *H. contortus* L3 obtaining 100% of larvicida activity, presumably because nematodes cuticle and eggs is constituted by chitin, been eggs who have higher levels^52^. *Serratia* spp was identified in gastric mucosa in our study, being the resistant group (0.12%) who had a higher abundance in comparison with the susceptible group (0.041%). This data may support the hypothesis that Serratia spp produces nematicidal compounds that collaborate in the control of the infection.

As a conclusion, our results suggest that resistant o susceptible phenotype to *T. circumcincta* infection could influence the microbiome composition, modifying the interaction with the host and in the last instance affecting the individual risk.

## Methods

### Ethical approval

All procedures involving animals in this study was performed in accordance to Spanish regulations regarding the protection of animals used for experimental and other scientific proposes (Royal Decree 53/2013), under the supervision of the Ethical and Animal Welfare Committee of University of León to after the approval of the competent body, Junta de Castilla y León.

All methods are reported in accordance with ARRIVE guidelines.

### Animals and experimental design

The description of this section was previously published by^53^. Briefly, a total of 18 adult ewes (6–8 years old) belonging to a Churra breed flock were selected after measuring the number of eggs per gram (epg) in faeces in 109 grazing animals naturally infected with GIN. Those animals with the highest and lowest FEC values were selected for subsequent deworming and experimental infection with a single dose of 50,000 T*. circumcincta* L3. Thirty days after this first infection, ewes with the lowest and highest cumulative FEC (cFEC), 6 resistant (mean cFEC: 308 ± 338 epg) and 6 susceptible (mean cFEC: 5594 ± 2661 epg) to the infection, were selected. The individual data related to cFEC are shown in supplementary material (Table S1)Then, the same ewes were infected again but in this case with a single dose of 70,000 T*. circumcincta* L3; at day 7 pi, all animals were humanly euthanized for the collection of the samples. At that moment of the infection, the resistant ewes had a L4 burden 68% lower than susceptible ones.

### Gastric content and abomasal tissue recovery

After sheep necropsy, the omaso and pylorus were tied using suture thread and immediately the abomasum was removed from all sheep. Abomasums were opened along the curvature and the inner surface was washed with tap water. : Both gastric contents and abomasal portions were immediately frozen in liquid nitrogen at the sampling site and then frozen at − 20 °C until use^35^.

### Microbial DNA extraction

Microbial DNA was extracted from abomasal gastric mucosa and gastric content from each animal. Abomasum portions were scraped to obtain the gastric mucosa sample using a sterile slide without excessive pressure keeping the samples on ice to avoid DNA degradation. Microbial genomic DNA was extracted using Purelink Genomic DNA mini-kit (Invitrogen; REF K182000, Spain)^38^.

Gastric content was lyophilized and homogenised. Then genomic DNA was extracted using QIAampPowerFecal Pro DNA Kit (Qiagen; REF 51,805, Germany)^54^.

Both kits were used in accordance with manufacturer’s instructions. After microbial DNA extraction, DNA was quantified using Nanodrop® ND-1000 Spectrophotometer.

### Total bacteria DNA

Total bacteria DNA was measured in all samples by quantitative real time PCR using forward primer (5’-GTG STG CAY GGY TGT CGT CA-3’) and reverse primer (5’- ACG TCR TCC MCA CCT TCC TC-3’) to calculate the number of copies in each samples, as previously described by^55^.

After extraction and quantification, samples were sent to amplify 16S rRNA hypervariable V3–V4 region. The sequencing was carried out by Teagasc Sequencing Centre (Moorepark, Fermoy, Ireland) service using 2 × 301 bp paired-end sequencing with Illumina MiSeq platform (Illumina, San Diego, CA, USA).

### Bioinformatic processing and statistical analysis

After quality control, the sequencing raw data was aligned against the sheep reference genome (Oar_rambouillet_v1.0,https://www.ensembl.org/Ovis_aries_rambouillet/Info/Index) to remove host DNA sequences. The retained sequences from the Fastq file were filtered and trimmed to 280 (forward) and 210 bp (reverse) using the filter and Trimm function of the DADA2 package^56^. The paired reads were assembled into Amplicon Sequences Variants (ASV) and their taxonomic assignment was performed using the SILVA nr v.138 database^57^. Richness analyses were performed in R V4.1. ASVs and variables (phenotype and type of sample) were included in the estimation of alpha diversity index (Chao1 Rarefied Species, Shannon’s Diversity index and Simpson Dominance index) using Phyloseq package from R. Normality was check using Shapiro–Wilk test and the homogenized of the variance was tested with Levene’s test. The differences between groups were estimated using Kruskal–Wallis. Beta diversity was plotted using Non-linear Multi-Dimensional Scaling (NMDS) to explore the dissimilarities between pairs of samples using Bray–Curtis dissimilarity index, and Unweighted Unifrac index using Vegan package from R software. The Vegan envfit function was used to evaluate if the factors of study (phenotype and sample type) where associated to the NMDS ordinations; the significance of the fitted factors was estimated by using 999 permutations.

Relative abundances were calculated for each sample. Normality was check using Shapiro–Wilk test. Then, differences between groups were estimated using U-Mann–Whitney. Correlation between cFEC levels and relative abundance of bacterial species was measured by Spearman coefficient. The level of significance was determinate at p < 0.05 and p values between 0.05–0.1 were considered approaching significance.

## Supplementary Information

Supplementary Information.

## Data Availability

All Illumina sequence data from the current study are available from the Sequence Read Archive (SRA) of NCBI (National Center of Biotechnology Information) under the BioProject ID PRJNA872890 (https://www.ncbi.nlm.nih.gov/sra/?term=PRJNA872890).
