# Supplementary material for: Microbial community in resistant and susceptible Churra sheep infected by Teladorsagia circumcincta
Source: Sci Rep. 2022 Oct 21;12:17620. doi: 10.1038/s41598-022-21058-x (PMC9587209; doi:10.1038/s41598-022-21058-x)
Supplement: Supplementary file 1 — Supplementary Information. [file 41598_2022_21058_MOESM1_ESM.docx]

Table S1: Cumulative faecal egg count (cFEC) values in egg per gram (epg) in faeces of each ewe after a first experimental infection. cFEC values was used to classify animals in both groups.
